# Correlates of inconsistent condom use and drug use among men having sex with men in Poland: a cross-sectional study

**DOI:** 10.1177/0956462420929136

**Published:** 2020-07-23

**Authors:** Krzysztof Piersiala, Joanna Krajewski, Daniela Dadej, Anna Loroch, Witold Czerniak, Błażej Rozpłochowski, Agata Kierepa, Iwona Mozer-Lisewska

**Affiliations:** 1 Student Research Group at the Department of Infectious Diseases, Hepatology and Acquired Immunodeficiencies, Karol Marcinkowski University of Medical Sciences, Poznań, Poland; 2 Division of ENT Diseases, Department of Clinical Sciences, Intervention and Technology, Karolinska Institute, Stockholm, Sweden; 3 Department of Infectious Diseases, Hepatology and Acquired Immunodeficiencies, Karol Marcinkowski University of Medical Sciences, Poznań, Poland

**Keywords:** Sexually transmitted infection, human immunodeficiency virus, men having sex with men, condom, drugs

## Abstract

The number of new human immunodeficiency virus (HIV) diagnoses is rising in many parts of Europe. We sought to evaluate the rising prevalence of new HIV diagnoses in Poland, where the majority of newly-diagnosed HIV cases are men having sex with men (MSM). This study aims to measure the prevalence of condom use and drug use and to identify risk factors for contracting sexually transmitted infections (STIs) among MSM in Poland by distributing an anonymous online survey aimed toward MSM. Among the 1438 participants who completed valid surveys, those with low education level and greater than 100 prior sexual partners showed the highest odds for inconsistent condom use (adjusted odds ratio [aOR] 3.027, 2.044, respectively). Participants who identified themselves as heterosexuals, with multiple sexual partners and living in big cities showed the highest odds for drug use (aOR 4.869, 3.305, 1.720, respectively). This study identifies groups at the highest risk of HIV/STIs and provides valuable information for public health experts to develop targeted STI prevention campaigns.

## Introduction

According to the World Health Organization human immunodeficiency virus (HIV)/acquired immune deficiency syndrome (AIDS) surveillance report in Europe, there have been 159,420 newly-diagnosed cases of HIV in 2017 in Europe, of which 25,353 were detected in the the European Union (EU), European Economic Area (EEA).^1^ Overall, there is a decreasing global trend of new HIV infections (by 16% since 2010); yet in some parts of Europe, the number of HIV diagnoses continues to rise.^2^ Sexual activity between men is the most common mode of transmission in Central Europe (accounting for 38.2% of diagnoses), and the ratio of HIV-positive males to females is 3.1 to 1.^1^ According to the National Institute of Public Health in Poland, between the years 1985 and 2018, there were 23,931 Polish citizens diagnosed with HIV, 3667 with AIDS and 1411 related deaths.^3^ Polish public health experts estimate that the majority of new cases are among men having sex with men (MSM); however, at diagnosis of HIV, the likely route of infection was unknown or incomplete in a high percentage of epidemiological reports (67.0%). This in turn limits development of targeted HIV prevention programs in Poland. More importantly, even though most European countries (including Poland) offer HIV testing and treatment free of charge, it is estimated that approximately 49% of cases are diagnosed at a late stage (defined as CD4 count<350 cells/mm^3^), which worsens their prognosis and increases the risk of HIV transmission.^1^ A combination of factors may contribute to the growing number of new HIV infections in MSM in Central Europe. Further study is needed to assess guidance by healthcare providers, sexual education and psychosocial stigma in the MSM population.

This study aims to measure the prevalence of condom use and drug use among MSM in Poland by distributing an anonymous online survey aimed toward MSM. This study also aims to identify risk factors for contracting sexually transmitted infections (STIs) such as socio-demographic information, sexual and high risk behavior, and drug use.

## Methods

### Study setting and participants

This national, cross-sectional study was conducted between November 2015 and April 2016 within Poland. Participants were recruited for this study using a standardized anonymous questionnaire sent via an online dating website aimed toward MSM. Eligibility for this study included male gender, ability to complete the questionnaire in Polish, one or more male sexual partners in the past and willingness to participate. The exclusion criteria included no history of male sexual partner, incomplete condom use and drug use questions and prior participation in the study.

### Sampling

A link to an anonymous questionnaire (created in Google Forms) was sent to 17,000 users of one of the most popular MSM dating website (https://www.fellow.pl) in Poland. This online dating platform was selected because according to results of Gemius/PBI internet audience research, Fellow.pl is particularly aimed at homosexual male users, and among various online dating platforms for MSM, it had the highest number of real users and views per year. The total number of users on this website as of April 2019 was 141,149. Initially, each subject was reached individually via their internal inbox associated with their profile. The study purpose and participation terms including anonymity, privacy and confidentiality were outlined. Next, only users who clearly stated willingness to participate in the study and agreed to the terms were sent a separate link to the survey. The response rate was 10.0%. In total, there were 1696 completed surveys of which 89 were excluded due to participants declaring not being sexually active, and 169 were excluded due to incomplete responses related to condom use and drug use.

### Measures

Participants were asked to complete a structured questionnaire in Polish. The development of the questionnaire was based on a review of literature. For the purpose of the study, high risk sexual behavior was defined as having unprotected anal intercourse (UAI) and/or having multiple sexual partners and/or drug use. The questionnaire had been pre-tested with 100 users of the same dating website and based on their feedback the study was refined. The final questionnaire consisted of 16 questions that were divided into three main sections including socio-demographics, sexual and high risk behavior and recreational or hard drug use. The subset of questions related to socio-demographics included: current self-identified gender (male or transgender) and sexual orientation (heterosexual, bisexual, homosexual, not listed above [please specify]), age (categorized in five groups), level of education (according to Polish educational system), place of residence (city population and region [voivodeship]). The subset of questions related to sexual and high risk behavior included: whether or not the participant currently has a long-term partner (defined as relationship longer than six months with one person), number of previous partners (categorized in five groups), whether or not the participant uses condoms during anal intercourse (AI) (with four possible answers: ‘always’ – considered as consistent condom use; ‘sometimes’, ‘never’ and ‘never with long-term partner’ – considered as inconsistent condom use). The subset of questions related to drug use included: recreational or hard drug use other than cannabinoids, and knowledge and attitudes toward STIs prophylaxis (not analyzed in this paper). The survey was self-reported and included single answer, multiple choice or rating scale questions. The anonymous questionnaire required logging into a Google account to ensure that each user participated in the questionnaire only once.

### Sample size calculation

Based on our pre-survey, a 52% prevalence value of consistent condom use during anal sex with other men among MSM was used for calculating the sample size, using the following formula: n = DEFF×Z2 1–α/2×P×(1–P)/d2, where n = minimum sample size required, DEFF = design effect, Z1–α/2 = z-score for the desired confidence level (1.96 for 95% confidence), P = estimated prevalence of consistent condom use and d = precision of 5% (0.05). For the purpose of the study, we assumed a design effect of 3.5 following recommendations of Johnston et al.^4^ Therefore, the minimum sample size was estimated to be 1341. We assumed that about 5% of the questionnaires would be incorrectly filled in or incomplete. For this reason, 67 more questionnaires (i.e. 0.05 × 1341) were added, giving a target of 1408 participants for the study sample size.

### Statistical analysis

Descriptive variables were summarized by n (%) for categorical variables. A univariate analysis was performed using the Fisher’s exact test for variables with less than or equal to five cases and the Chi square test for variables with greater than five cases. A p-value of <0.05 was considered statistically significant. Logistic regression was used to evaluate association between sets of descriptive variables such as consistent condom use and drug use. The variables with statistical significance of p < 0.05 in the univariate analysis were included in the multivariate logistic regression model. Estimates of odds ratio (OR), 95% confidence interval (CI) and p-values are presented for all analyses. All calculations were performed with use of IBM SPSS Statistics 25 (IBM Corp., Armonk, NY, USA).

### Ethics

The study protocol was approved by the Bioethics Committee of Poznan University of Medical Sciences (approval no. 1071/71). The study was performed in accordance with the ethical standards of the institutional Bioethics Committee of the Poznan University of Medical Science, and with the 1964 Helsinki declaration and its later amendments or comparable ethical standards. The research did not include clinical trials.

## Results

### Patient characteristics

At the completion of the study, 1438 valid completed surveys were obtained. Table 1 summarizes the socio-demographic characteristics of the participants. Overall, 79.1% of the respondents were aged between 18 and 34 years and 47.0% declared living in cities with greater than 100,000 inhabitants. Both Eastern and Western Poland were nearly equally represented with 49.2% of participants living in Eastern Poland and 50.8% living in Western Poland. The majority of participants (60.2%, n = 865) reported completing higher education and 8.8% reported completing primary or vocational education. The majority of participants identified themselves as homosexual (75.5%), followed by bisexual (20.1%) and heterosexual (4.5%). Among the participants, 29.6% (n = 426) declared being in a relationship lasting longer than six months, while 43.5% declared having had two to nine sexual partners in the past.

**Table 1.** Socio-demographic characteristics of respondents.

Variable	Number of respondents (N = 1438)	Percentage (%)
Age category (years)		
<18	51	3.5
18–24	679	47.2
25–34	459	31.9
35–45	143	9.9
>45	106	7.4
Size of city (population)		
<5000	249	17.3
5000–20,000	159	11.1
20,000–100,000	354	24.6
>100,000	676	47.0
Education		
Primary education	69	4.8
Secondary education	446	31.0
University education	865	60.2
Vocational education	58	4.0
Sexual orientation		
Homosexual	1085	75.5
Bisexual	289	20.1
Heterosexual	64	4.5
Currently in relationship		
Yes	426	29.6
No	1012	70.4
Number of sexual partners		
1	151	10.5
2–9	625	43.5
10–24	348	24.2
25–100	232	16.1
>100	82	5.7
Part of Poland		
Eastern	708	49.2
Western	730	50.8

### Condom use

Characteristics of condom use during AI among Polish MSM are shown in Table 2. Just over half of the respondents, 50.5% (n = 736), declared consistent condom use (always) during AI. The following three variables: education level, relationship status and number of sexual partners in the past show significant differences between subgroups (p = 0.001, p < 0.001 and p = 0.023, respectively).

**Table 2.** Results of univariate and multivariate logistic models with consistent condom use as dependent variable.

Variable	Consistent condom use of participants		Chi square test (p-value)	Univariate model		Multivariate model	
	YesN = 726 (50.5%)	NoN = 712 (49.5%)		OR (95%CI)	p-Value	aOR (95%CI)	p-Value
Age category (years)							
<18	24 (47.1)	27 (52.9)	0.428	Ref.	–	–	–
18–24	350 (51.5)	329 (48.5)		0.836 (0.472–1.478)	0.537		
25–34	239 (52.1)	220 (47.9)		0.818 (0.458–1.461)	0.497		
35–45	63 (44.1)	80 (55.9)		1.129 (0.594–1.461)	0.711		
>45	50 (47.2)	56 (52.8)		0.996 (0.510–1.944)	0.990		
Size of city (population)							
<5000	120 (48.2)	129 (51.8)	0.411	Ref.	–	–	–
5000–20,000	74 (46.5)	85 (53.5)		1.069 (0.717–1.592)	0.745		
20,000–100,000	176 (49.7)	178 (50.3)		0.941 (0.680–1.301)	0.712		
>100,000	356 (52.7)	320 (47)		0.836 (0.625–1.118)	0.228		
Education							
University education	457 (52.8)	408 (47.2)	0.001	Ref.	–	Ref.	
Primary education	30 (43.5)	39 (56.5)		1.456 (0.888–2.387)	0.136	1.736 (1.037–2.906)	0.036
Secondary education	223 (50.0)	223 (50.0)		1.120 (0.891–1.408)	0.331	1.249 (0.985–1.584)	0.067
Vocational education	16 (27.6)	42 (72.4)		2.940 (1.628–5.310)	<0.001	3.027 (1.655–5.538)	<0.001
Sexual orientation							
Homosexual	542 (50.0)	543 (50.0)	0.098	Ref.		–	–
Bisexual	158 (54.7)	131 (45.3)		0.828 (0.638–1.074)	0.154		
Heterosexual	26 (40.6)	38 (59.4)		1.459 (0.874–2.436)	0.149		
Currently in relationship							
No	552 (54.5)	460 (45.5)	<0.001	Ref.	–	Ref.	–
Yes	174 (40.8)	252 (59.2)		1.738 (1.382)	<0.001	1.700 (1.348–2.145)	<0.001
Number of sexual partners							
1	95 (62.9)	56 (37.1)	0.023	Ref.		Ref.	–
2–9	306 (49.0)	319 (51.0)		1.768 (1.227–2.549)	0.002	1.923 (1.319–2.805)	0.001
10–24	176 (50.6)	172 (49.4)		1.658 (1.121–2.452)	0.011	1.880 (1.250–2.828)	0.002
25–100	108 (46.6)	124 (53.4)		1.948 (1.281–2.962)	0.002	2.243 (1.446–3.478)	<0.001
>100	41 (50.0)	41 (50.0)		1.696 (0.984–2.924)	0.057	2.044 (1.167–3.580)	0.012
Part of Poland							
Western	362 (49.6)	368 (50.4)	0.489	Ref.	–	–	–
Eastern	364 (51.4)	344 (48.6)		0.930 (0.756–1.143)	0.489		
HIV status							
HIV(+)	23 (53.5)	20 (46.5)	0.690	Ref.		–	–
HIV(–)	703 (50.4)	692 (49.6)		1.132 (0.616–2.080)	0.690		

Vocational education was associated with inconsistent condom use compared to other educational levels (27.6% vs. 51.4%, p = 0.001). Participants with a vocational education had increased odds of inconsistent condom use compared to participants with a university education (OR 2.940, 95%CI 1.628–5.310, p < 0.001). Multivariate analysis adjusted for the number of sexual partners and relationship status showed that vocational education and primary education were independent factors associated with inconsistent condom use (aOR 3.027 95%CI 1.655–5.538, p < 0.001 for vocational education and aOR 1.736, 95%CI 1.037–2.906, p = 0.036 for primary education) (Table 2).

Participants in a long-term relationship showed a significantly lower rate of consistent condom use compared to those that were single (40.8% vs. 54.5%, p < 0.001). The odds for inconsistent use of condom were significantly increased for those in a relationship (OR 1.738, 95%CI 1.382–2.186, p < 0.001). This association persisted and showed statistical significance by multivariate analysis (aOR 1.700, 95%CI 1.348–2.145, p < 0.001) (Table 2).

When assessing the association between condom use and number of sexual partners, respondents declaring just one sexual partner in the past had the highest rate of consistent condom use (62.9% vs. 46.6%–50.6% in remaining subgroups, p = 0.023). All subgroups with a number of sexual partners greater than one showed increased odds for inconsistent use of condoms compared to the reference (one sexual partner) – (OR 1.768, 95%CI 1.227–2.549, p = 0.002 for 2–9 partners, OR 1.658, 95%CI 1.281–2.962, p = 0.011 for 10–24 partners, OR 1.948, 95%CI 1.281–2.962, p = 0.002 for 25–100 partners and OR 1.696, 95%CI 0.984–2.924, p = 0.057 for >100 partners). All subcategories remained statistically significant during multivariate analysis (Table 2).

### Drug use among Polish MSM

Findings related to drug use among MSM are presented in Table 3. Fourteen percent of respondents (n = 205) declared recreational or regular use of drugs (other than cannabis). The majority (86.8%, n = 178) declared using drugs rarely and the remainder (13.2%, n = 27) declared using drugs often. Of the participants declaring drug use, 22.9% (n = 47) had sex with a casual partner under the influence of drugs. Drug use was strongly associated with the number of sexual partners, ranging from 8.6% for one sexual partner to 24.4% for over 100 sexual partners (p < 0.001). Those with 10–24, 25–100 and >100 sexual partners had an increased odds ratio of drug use compared to the reference (one sexual partner) (OR 1.907, 95%CI 1.006–3.615, p = 0.048, OR 2.916 95%CI 1.524–5.581, p= 0.001 and OR 3.424 95%CI 1.602–7.321, p = 0.001, respectively). These remained statistically significant during multivariate analysis (Table 3).

**Table 3.** Results of univariate and multivariate logistic models with drug use as dependent variable.

Variable	Participants using drugs		Chi square test(p-value)	Univariate model		Multivariate model	
	YesN 205 (14.3%)	NoN= 1233 (85.7%)		OR (95%CI)	p-Value	aOR (95%CI)	p-Value
Age category (years)							
<18	9 (17.6)	42 (82.4)	0.481	Ref.	–	–	–
18–24	106 (15.6)	573 (84.4)		0.863 (0.408–1.826)	0.701		
25–34	61 (13.3)	398 (86.7)		0.715 (0.332–1.543)	0.393		
35–45	18 (12.6)	125 (87.4)		0.672 (0.281–1.609)	0.672		
>45	11 (10.4)	95 (89.6)		0.540 (0.208–1.401)	0.205		
Size of city (population)							
<5000	24 (9.6)	225 (90.4)	<0.001	Ref.	–	Ref.	
5000–20,000	13 (8.2)	146 (91.8)		0.835 (0.412–1.692)	0.616	0.863 (0.422–1.766)	0.687
20,000–100,000	44 (12.4)	310 (87.6)		1.331 (0.786–2.252)	0.287	1.240 (0.725–2.123)	0.432
>100,000	124 (18.3)	552 (81.7)		2.106 (1.324–3.349)	0.002	1.720 (1.062–2.783)	0.027
Education							
Vocational education	8 (13.8)	50 (86.2)	0.347	Ref.	–	–	–
Primary education	13 (18.8)	56 (81.2)		1.451 (0.556–3.788)	0.447		
Secondary education	54 (12.1)	392 (87.9)		0.861 (0.387–1.914)	0.713		
University education	130 (15.0)	735 (85.0)		1.105 (0.512–2.386)	0.798		
Sexual orientation							
Homosexual	148 (13.6)	937 (86.4)	<0.001	Ref.	–	Ref.	–
Bisexual	34 (11.8)	255 (88.2)		0.844 (0.567–1.256)	0.404	0.914 (0.609–1.372)	0.666
Heterosexual	23 (35.9)	41 (64.1)		3.552 (2.071–6.090)	<0.001	4.869 (2.720–8.714)	<0.001
Currently in relationship							
No	159 (15.7)	853 (84.3)	0.015	Ref.	–	Ref.	–
Yes	46 (10.8)	380 (89.2)		0.649 (0.458–0.921)	0.016	0.563 (0.391–0.810)	0.002
Number of sexual partners							
1	13 (8.6)	138 (91.4)	<0.001	Ref.	–	Ref.	–
2–9	69 (11.0)	556 (89.0)		1.317 (0.708–2.452)	0.384	1.472 (0.772–2.807)	0.240
10–24	53 (15.2)	295 (84.8)		1.907 (1.006–3.615)	0.048	2.103 (1.073–4.121)	0.030
25–100	50 (21.6)	182 (78.4)		2.916 (1.524–5.581)	0.001	3.305 (1.661–6.576)	0.001
>100	20 (24.4)	62 (75.6)		3.424 (1.602–7.321)	0.001	3.138 (1.413–6.966)	0.005
Part of Poland							
Western	105 (14.4)	625 (85.6)	0.888	Ref.	–	–	–
Eastern	100 (14.1)	608 (85.9)		0.979 (0.728–1.316)	0.888		
Consistent condom use							
No	109 (15.3)	603 (84.7)	0.258	Ref.	–	–	–
Yes	96 (13.2)	630 (86.6)		0.843 (0.627–1.134)	0.258	–	–
HIV status							
HIV(–)	195 (14.0)	1200 (86.0)	0.087	Ref.			
HIV(+)	10 (23.3)	33 (76.7)		1.865 (0.905–3.845)	0.091		

HIV: human immunodeficiency virus; OR: odds ratio; CI: confidence interval; aOR: adjusted odds ratio.

We observed that the number of inhabitants correlated with drug use as follows. In cities smaller than 20,000 inhabitants, under 10% of participants declared drug use compared to 18.3% in cities with greater than 100,000 inhabitants (p < 0.001). Those living in cities greater than 100,000 inhabitants had significantly increased odds of drug use compared to others (OR 2.106, 95%CI 1.324–3.349, p = 0.002). This association remained statistically significant on multivariate analysis (aOR 1.720, p = 0.027) (Table 3).

We also observed that sexual orientation was associated with drug use among respondents. Those who identified themselves as heterosexual (35.9%) reported drug use significantly more often (13.6% for homosexual and 11.8% for bisexual, p < 0.001). Heterosexual MSM had significantly increased odds of drug use compared to homosexual MSM (OR 3.552, 95%CI 2.071–6.090, p < 0.001). Being in a relationship longer than six months showed decreased odds of drug use (OR 0.563, 95%CI 0.458–0.921, p = 0.016). Statistical significance was again noted on multivariate analysis (aOR 0.563, 95%CI 0.391–0.810, p = 0.002). We did not observe any significant correlation with age, education level, region of Poland and consistent use of condoms with drug use (Table 3).

## HIV status in relation to high risk sexual behaviors

In the studied cohort, 43 (3.0%) respondents declared that they were living with HIV. HIV status did not correlate with consistent condom or drugs use. However, there was a trend toward drug use with HIV-positive status, although this was not statistically significant (23.3% vs. 14.0%, p = 0.087, respectively). Results are summarized and presented in Tables 2 and 3.

## Discussion

In our study, we observed that high risk-taking behaviors such as inconsistent condom use and drug use occur among MSM in Poland. Currently, most of the available research concerning the Polish MSM population is based on participants seeking medical help in relation to STI testing or diagnosis. With this study, we managed to reach beyond the MSM population seeking medical help/counseling regarding their sexual health. To our knowledge, we are the first to investigate unprotected AI and drug use among internet users of the MSM population in Poland. Within our cohort, we found that 50.5% of participants declared consistent condom use (always) when having AI. The groups with the highest odds of inconsistent condom use included those with a low education level and those declaring more than 100 sexual partners in the past. Drug use was associated with an increased number of sexual partners, larger city size and heterosexual orientation.

One of the strongest risk factors for HIV in the MSM population is UAI, which is an indicator of overall high risk sexual behavior.^5^ Hibbert et al. highlighted three categories associated with sexual risk-taking behaviors: individual (demographic, mental health, personal beliefs), interpersonal (relationship status, intimacy issues, interpersonal communication) and situational/societal.^6^ In our study, we identified that the demographic predictors of condom use during AI include educational level, relationship status and number of previous sexual partners. In line with our results, having a steady partner was described to be associated with inconsistent condom use,^7^ additionally a negative correlation with consistent condom use was seen among young internet users and MSM living with HIV.^5^ In contrast to our results, the association between educational level and UAI in literature is ambiguous.^7,8^

In light of the increasing number of STIs in Europe, especially among MSM,^9^ a comparison was made to other areas of the world to assess for similar trends of high risk sexual behavior and drug use. Johansson et al. assessed high risk-taking sexual behaviors in Sweden, similarly finding that low education, inhabitants of metropolitan areas and being single were all associated with higher rates of unprotected AI.^10^ In France, financial hardship was listed as a factor contributing to engagement in unprotected AI and transactional sex increased the possibility of STI transmission.^11^ Swiss MSM appeared to use condoms less often when depressed or when engaging with HIV-positive partners.^12^ Brazilian MSM with the highest scores of high risk behavior included age below 25, becoming sexually active prior to 15 years of age, finding partners on the internet, and frequent alcohol and illicit drug use.^13^ In China, on the other hand, the most likely group to engage in unprotected sexual activity was a population of MSM older than age 36 who would most commonly meet their sexual partners in public parks.^14^

We investigated unprotected AI among internet users in the MSM population in Poland. We observed that there was less consistent use of condoms among those in a long-term relationship. We acknowledge that some authors^7^ suggest that condom non-use in seroconcordant monogamous relationship may be appropriate for MSM. However, in our paper, we decided to categorize this behavior as high risk due to the low rates of testing for HIV in the lesbian, gay, bisexual, and transgender (LGBT) community in Poland compared to Western countries such as USA or Western Europe. Likely in part due to the ongoing stigma associated with being part of the LGBT community and getting tested in Poland, we believe that a large proportion of participants may assume seroconcordance in their relationship and thus, we defined unprotected AI as high risk sexual behavior. Additionally, inconsistent condom use was observed in about 50% of those declaring a high number of sexual partners, thus increasing the risk of transmission to multiple partners, likely unaware of their HIV status. Therefore, the promotion of consistent condom use should be addressed in new campaigns.

Drug use is also an accepted indicator of high risk sexual behavior. Duan et al. noticed a positive correlation between recreational drug use in the Chinese MSM population (rush poppers being the most popular) and a higher risk of HIV/STI transmission.^15^ The same trend was reported by Marcus et al. who assessed 13 European cities, where the prevalence of HIV in drug-using and non drug-using populations was 10.5% vs. 3.9%, respectively.^16^ In our study, we identified several risk factors associated with more frequent drug use among Polish MSM. The risk factors we observed include living in big cities, identifying as heterosexual and having multiple partners. Our results are congruent with the study performed in the UK by Hibbert et al.,^6^ where sexualized drug use was associated with living in a densely populated city, a higher number of sexual partners (which were also trends noted in our study) and recent STI diagnosis, as well as low life satisfaction.^6^ An American paper on adolescent and young adult MSM similarly showed that individuals with a high number of casual partners were more likely to exhibit high risk sexual behaviors, such as alcohol/substance use prior to sexual activity.^17^

A recent study, performed among British MSM in 2019, indicates an increased prevalence of substance use among MSM in comparison to the general population.^6^ The percentage of recreational and hard drugs users in Poland (according to the National Report of 2018 prepared by the National Bureau for Drug Prevention) is estimated at 4.7% (data for people aged 15–64). The declared prevalence of drug use in our sample was approximately three times higher compared to the estimate reported above. Drug use in Poland is observed mainly among young adults, especially those aged 25–34.^18^ Although we did observe a similar trend, the differences between age groups did not reach statistical significance, suggesting that the increased use of drugs applies to the entire group, including middle-aged and senior MSM. In contrast to other publications, our study did not show a significant correlation between the use of psychoactive substances and UAI. However, there was a trend toward high risk-taking behaviors in the group declaring drug use. In our sample, sex under the influence of drugs was relatively low (3.3%) compared to the aforementioned study of Hibbert et al. Even though the drug use is associated with higher number of sexual partners in our study, it does not necessarily correlate with UAI among Polish MSM. Another unexplored field is the population of MSM identifying themselves as heterosexuals. In our study, this group showed high risk behaviors when it comes to sexual intercourse between men. In our sample, they had both increased odds for UAI and drug use compared to homosexual and bisexual MSM. This group of MSM in Poland may identify themselves as heterosexuals, possibly due to social stigma, and their high risk sexual behavior may not be adequately addressed. This group may not typically visit LGBT places such as bars, restaurant or events where most of the preventive campaigns take place. These findings support that this group should be addressed in future safe sex and prevention campaigns. One solution is to try to reach them by means of Internet and dating websites.

Several limitations are observed in Poland’s sexual education curriculum. For example, the sexual education program does not discuss or include homosexuality as one of the topics, therefore omitting MSM-related sexual health issues and dangers. Our study shows that high risk behaviors are common among Polish MSM irrespective of age. One of the major causes of persisting stigma toward homosexuality in Poland is thought to be related to ongoing political disputes. As a result, MSM do not receive adequate information regarding maintaining a healthy sexual lifestyle and it is challenging to assess their source of knowledge on safe sex practices. This includes assessing the knowledge of sexual education teachers and primary care physicians. Another underestimated issue in Polish HIV prevention campaigns is MSM who are in relationship with other men (28% of the studied cohort according to our study). In particular, HIV serodiscordant couples are not covered for pre-exposure prophylaxis (PrEP) by Poland’s national health insurance.

Our study has several limitations. Firstly, the online accessibility of the survey results in a selection bias; therefore, the results cannot be generalized to MSM individuals in Poland not utilizing online dating platforms. However, we believe that a vast majority of sexually active MSM utilize Internet dating and social websites actively; thus, it was a convenient route to reach a representative sample of this group. There is information bias with the survey format due to the inherent intimate and personal nature of the questions included in the survey. This may lead to failure in providing accurate answers and our analysis is dependent on the reliability of these responses. However, we believe that the anonymous and online format of this survey helps to achieve a higher answer accuracy than face-to-face contact with an investigator. In the questionnaire, we asked about the use of psychoactive substances (other than cannabis); however, another limitation is that we did not specifically ask the participants to enter the name of the drugs they use or used in the past. Furthermore, in our study methodology, we considered the answer ‘never with long-term partner’ as an inconsistent condom use. Lastly, the accuracy of some responses could be influenced by recall bias.

Despite these limitations, we believe that our study has clear strengths and provides interesting observations which are listed below:

1. It reveals that, despite of HIV prevention campaigns in Poland, the use of condoms among MSM in Poland is low. The low rate of condom use is not related to PrEP use, which as of April 2019 remains unavailable in Poland.
2. It identifies the highest risk groups among Polish MSM: those with lower education level and those with multiple sexual partners.
3. To the best of our knowledge, this is the first study reporting a high percentage of MSM in Poland living in long-term relationships (29.6%). This information should be taken into account when developing new targeted prevention campaigns.
4. It identifies a higher rate of drug use among Polish MSM compared to the general population.

## Conclusion

The use of condoms during AI is low among Polish MSM. Those with a lower education level and those with multiple sexual partners are at the highest risk of inconsistent condom use. A substantial fraction of MSM in Poland declares regular use of drugs. This study provides valuable information for public health experts in Poland and Eastern Europe to help tailor targeted HIV and STI prevention programs.
